# Serum levels of interleukin-33 and mesencephalic astrocyte derived neurotrophic factors in patients with major depressive disorder: a cross-sectional comparative design

**DOI:** 10.1186/s12888-023-05463-8

**Published:** 2024-01-12

**Authors:** Zabun Nahar, Delruba Tabassum Nowshin, A. S. M. Roknuzzaman, Md. Sohan, Salsabil Islam, M. M. A. Shalahuddin Qusar, Md. Rabiul Islam

**Affiliations:** 1 Department of Pharmacy, University of Asia Pacific, 74/A Green Road, Farmgate, Dhaka 1205 Bangladesh; 2 Department of Psychiatry, Bangabandhu Sheikh Mujib Medical University, Shahabagh, Dhaka 1000 Bangladesh; 3 School of Pharmacy, BRAC University, KHA 224, Progati Sarani, 1212 Merul Badda, Dhaka Bangladesh

**Keywords:** Major depressive disorder, Interleukin-33, IL-33, Mesencephalic astrocyte derived neurotrophic factor, MANF, ELISA, Cytokines, Comparative design, Cross-sectional study

## Abstract

**Background:**

Major depressive disorder (MDD) is a debilitating health condition that has significant morbidity and mortality rates. Depression can be caused due to social, biological, environmental, psychological, and genetic factors. A few biological processes have been proposed as the pathophysiological pathways of depression. Neurotrophic factors and inflammatory cytokines have been linked to depression. Thus, we aimed to investigate the serum interleukin-33 (IL-33) and mesencephalic astrocyte-derived neurotrophic factor (MANF) in MDD patients and corresponding healthy controls (HCs).

**Method:**

This study involved the inclusion of 129 MDD patients and 125 HCs matched by sex and age. A psychiatrist evaluated the study participants following DSM-5 criteria. The severity of the illness was assessed utilizing the Hamilton Depression Rating Scale (Ham-D). The serum concentrations of IL-33 and MANF were measured using enzyme-linked immunosorbent assay (ELISA) kits.

**Results:**

The mean serum levels of IL-33 were decreased (159.12 ± 6.07 pg/ml vs. 180.60 ± 8.64 pg/ml, p = 0.042), and the MANF levels were increased (5.40 ± 0.19 ng/ml vs. 4.46 ± 0.21 ng/ml, p = 0.001) in MDD patients when compared to HCs.

**Conclusions:**

The current study proposes that lower IL-33 and higher MANF serum levels are associated with MDD progression and depression severity. These biomarkers could be used as risk assessment tools for MDD. We recommend more investigation, including a significant population, to determine the precise function of IL-33 and MANF in depression.

## Background

Major depressive disorder (MDD) is a mental health status with persistent low mood and loss of interest in doing regular activities. Depression results from intricate interactions between environmental, biological, genetic, and epigenetic factors [1–5]. The development of MDD is also influenced by biological, social, and psychological variables [6–9]. According to the DSM-5, individuals have MDD if they display depressive, noticeable symptoms daily for at least two weeks or more. These are low mood, decreasing interest or enjoyment, appetite, weight, concentration changes, sleeping problems, lack of energy, guilty feelings, and recurrent suicidal thoughts [10–12]. It is a disease of primary care that goes undiagnosed far too frequently. According to National Institutes of Mental Health (NIMH) surveys, 70% of depressed people do not receive medical treatment for their condition. In later life, depression is a frequent illness linked to more extended periods of incapacity, higher costs, and worsening health outcomes [13]. Patients with depression have a higher level of functional impairment than people with chronic medical conditions like hypertension, diabetes, arthritis, or coronary artery disease [14]. Globally, MDD impacts 350 million people and is projected to become a leading cause of functional disability. It affects approximately 6% of the world’s population annually, with a higher prevalence in women [15–17]. In China and South Korea, MDD rates are 2% and 6.7%, contrasting with 21% in Chile and France. Europe shows the highest MDD prevalence, while Asia has the lowest [18–21]. In Bangladesh, a 2003–2005 survey disclosed MDD affecting 4.6% of adults, with a 45% higher likelihood of women lacking childcare, outside jobs, or alternative income sources [22–23].

Numerous biological processes have been identified, including alteration in systems related to dopamine, noradrenaline, serotonin, and glutamate, alteration in the hypothalamic-pituitary-adrenal (HPA) axis, an increase in inflammation, vascular alterations, along with impaired neuroplasticity, which are some potential pathophysiological causes of depression [24–27]. These suggested processes all interact with one another and are closely connected. One important concept for the pathogenesis of depression is the monoamine hypothesis, which claims that depression eventually results from alterations in the amounts of at least one monoamine, i.e., norepinephrine, serotonin, and dopamine [28]. Also, it has been discovered that in the progression of depressive disorders, neurotrophic factors play a part [29]. Moreover, researchers have looked into the fundamental processes through which cytokines may influence depression. In general, it has been shown that cytokines may reach the brain and interfere with almost all pathophysiological aspects of depression. Nuclear factor kappa B (NF-κB) was discovered as a crucial mediator at the blood-brain barrier, which could potentially communicate with the peripheral inflammatory signals of the central nervous system (CNS). According to a study, specific cell types can be activated by peripheral cytokine signals that enhance central inflammatory responses in the human brain. MDD and other neuropsychiatric diseases have been included in the recognition that inflammation could be the reason behind the development of these diseases. Increased inflammatory cytokines and other peripheral blood biomarkers were observed in MDD patients [30]. Increased inflammation does not occur in every MDD patient, indicating that those who belong to certain depressed groups, such as those who have pre-existing cognitive and genetic vulnerabilities or histories of trauma, may exhibit MDD caused by inflammation [31].

A few studies have been conducted to determine the relationship between interleukin-33 (IL-33) and MDD. According to several studies, the serum IL-33 levels in depressive and other psychiatric disorder patients were higher when compared to healthy controls (HCs) [31, 32]. Another study concluded that the serum levels of IL-33, a pro-inflammatory cytokine, were elevated in depressive episodes [33]. An increase in neuroimmune gene expression occurred due to early-life stress. Such stress involves astrocytes and endothelial cells secreting IL-33. This cytokine was found to interfere with a heterodimeric complex consisting of interleukin-1 receptor accessory protein (IL-1RAcP) and tumorigenicity 2 (ST2). ST2 receptor to encourage the production of chemokines and other cytokines. By altering the effect of regulation-related neural circuits, IL-33 may directly or by instigating the production of MDD risk moderating chemokines or cytokines, IL-33 may indirectly affect the risk of MDD [31].

On the other hand, mesencephalic astrocyte-derived neurotrophic factor (MANF) is one form of the novel neurotrophic factor and has been found to have cytoprotective effects in neurological disorders. MANF can shield cells against endoplasmic reticulum (ER) stress, as demonstrated by several studies [34]. ER stress is the condition when unfolded proteins accumulate in the ER lumen, inducing pro-inflammatory responses and, ultimately, apoptotic cell death. Excessive ER stress plays a role in developing many diseases, including neurodegenerative disorders. A crucial transcriptional regulator known as NF-κB plays a vital role at the beginning of inflammation after IκB degradation. By blocking p65 from adhering to the promoter of its target genes, MANF suppresses the NF-κB signaling cascade. MANF constantly reduces the expression of NF-κB dependent genes. Therefore, by interacting with p65, MANF may be a unique suppressor of inflammation [35]. As it has already been proven that stress can act as a potential risk factor for depression and induce inflammation [36], by negatively inducing inflammation, MANF may reduce depression risk. According to the results of a separate study, in contrast to HCs, MDD patients exhibited a reduced MANF level [37].

Thus, it still needs to be understood how IL-33 and MANF affect the development and course of MDD. Due to the inconclusiveness of findings regarding the role of IL-33 and MANF in MDD, we intended to determine the serum levels of these biomarkers in MDD patients and HCs. Moreover, the severity of depression would be measured by the altered IL-33 and MANF levels, if any.

## Methods

### Study design and participants

This study is mainly designed as a cross-sectional study with comparative groups that recruited cases and controls from October 1, 2022, to November 30, 2022. The MDD patients were selected from the Psychiatric Department of Bangabandhu Sheikh Mujib Medical University, Bangladesh, while all HCs were selected from various parts of Dhaka. Our target populations were adult males and females aged 18 to 60. We included 129 MDD patients and 125 HCs matched for sex and age. The eligibility criteria for enrollment encompassed individuals who had been evaluated by an experienced professional in the field of psychiatry. Additionally, the clinical interviews administered by psychiatrists adhered to the diagnostic guidelines outlined in the DSM-5, ensuring a standardized and comprehensive approach to the assessment process. The Hamilton Depression Rating Scale (Ham-D) was used to assess the severity of depression. In a 17-items Ham-D rating scale, scores of 0–7, 8–13, 14–18, 19–22, and 23 or more are regarded as normal, mild depression, moderate depression, severe depression, and very severe depression, respectively [38]. The inclusion criteria for this study comprised MDD patients who had had depressive symptoms for at least two weeks and did not use any drug regarding those symptoms. The use of alcohol, unprescribed drugs during the previous six months, cardiac, hepatic, kidney, inflammatory disease history, and severe somatic disorders were all considered exclusion factors. We included those participants who did not take any antidepressant or antipsychotic medications for at least a week that may affect serum IL-33 and MANF levels. The study excluded pregnant people and those with comorbidities with other psychotic diseases. As a result, patients with other AXIS I diseases were also excluded. Patients with mutism, non-participation, or cognitive impairment were not allowed to participate in the study. Similar to our previous studies, a pre-structured questionnaire was employed for collecting the sociodemographic details of the research subjects [4, 7].

### Blood sample collection, processing, and storage

A 5 ml blood sample was taken using the standard blood sampling techniques for additional laboratory testing. Blood was kept standing for one hour in a falcon tube to get clotted, then centrifuged at 1000 g for 15 min at 25^o^C to separate serum samples from blood samples. After centrifugation, serum was taken in an Eppendorf tube and stored at -80 °C. Commercially available Human IL-33 Picokine ELISA kits and Human MANF PicoKine ELISA kits (Boster Bio, USA) were used to determine the serum levels of IL-33 and MANF, respectively, for further analysis.

### Analysis of samples

The entire procedure was carried out per the manufacturer’s instructions. First, we removed microplate strips from the plate frame, and then we filled the appropriate wells on a 96-well microplate with 100 µl each of the sample and standard solutions. After that, the plate was covered with plate sealer, and the plates were incubated for 120 min at 25 °C. Then, we removed the cover from the plates and discarded the liquids. Then, we added 100 µl of detection antibody for the respective cytokine to the specific wells. After sealing with plate sealer, we left the plates for incubation for 60 min at 37 °C. Then, the contents of each well were discarded, and 300 µl of wash buffer was used for three rinses. After that, 100 µl of the avidin-biotin-peroxidase complex was added to each well, and then all the plates were incubated at 25 °C for 40 min. After discarding the liquids, each plate was washed five times in a row using 300 µl of wash buffer. Before leaving the plates for incubation in the dark at room temperature for 30 min, 90 µl of the color-developing agent was added to each well. Lastly, 100 µl of stop solution was added to complete the process. At last, absorbance was measured at 450 nm immediately. We calculated the serum IL-33 level as pg/ml and the serum MANF level as ng/ml.

### Statistical analysis

Microsoft Excel 2019 and IBM SPSS version 25.0 were utilized for data processing and analysis, respectively. In order to compare the study’s parameters between study groups, an independent sample t-test and a chi-square test were used. Also, associations among serum levels of biomarkers and clinical variables were analyzed by Pearson correlation coefficient analysis. The differences in serum IL-33 and MANF levels between the groups were shown by error bar graphs. Besides, in order to demonstrate the relationships between IL-33 and MANF levels in the serum and Ham-D scores, we constructed scatter plot graphs. A significant p-value was set at p < 0.05.

## Results

### Characteristics of the study participants

We presented the sociodemographic characteristics of all the study subjects in Table 1. Female MDD patients constituted 73.65%, with 26.35% being male; in HCs, females were 73.60%, and males were 26.40%. Predominantly in both groups, participants were between the ages of 18 and 25 (38.76% and 37.60%, respectively). Regarding educational background, 41.87% of patients completed the secondary level, while 46.40% of HCs graduated. In this study, most patients and HCs lived in urban areas. Compared to HCs (68.80%), married participants were in a higher proportion of MDD patients (68.22%). Most study participants belonged to the medium economic class (62.02% MDD patients and 57.60% HCs). Notably, 39.53% of MDD patients were housewives, 51.94% of patients had dealt with MDD before, and 75.19% had no family history of MDD. Nonsmokers were predominant in both groups (93.02% patients, 96.00% HCs), with over 50.00% having a normal BMI.

**Table 1:** Socio-demographic profile of the study population

Characteristics	MDD patients (n = 129) Mean ± SEM	Healthy controls (n = 125) Mean ± SEM	*p-value*
Age in years	30.67 ± 0.86	30.18 ± 0.82	0.675
18–25	50 (38.76%)	47 (37.60%)	
26–35	42 (32.56%)	42 (33.60%)	
36–45	28 (21.71%)	28 (22.40%)	
46–60	9 (6.97%)	8 (6.40%)	
Sex			0.105
Male	34 (26.35%)	33 (26.40%)	
Female	95 (73.65%)	92 (73.60%)	
Marital Status			0.873
Married	88 (68.22%)	81 (64.80%)	
Unmarried	41 (31.78)	44 (35.20%)	
BMI (kg/m^2^)	23.44 ± 0.44	24.38 ± 0.37	0.091
Below 18.5 (CED)	18 (13.96%)	5 (4.00%)	
18.5–25 (normal)	68 (52.71%)	63 (50.40%)	
Above 25 (obese)	43 (33.33%)	57 (45.60%)	
Education level			0.164
No formal education	6 (4.65%)	7 (5.60%)	
Primary level	24 (18.60%)	12 (9.60%)	
Secondary level	54 (41.87%)	58 (46.40%)	
Graduate and above	45 (34.88%)	48 (38.40%)	
Occupation			0.307
Business	7 (5.43%)	4 (3.20%)	
Service	17 (13.18%)	25 (20.00%)	
Housewife	51 (39.53%)	39 (31.20%)	
Unemployed	33 (25.58%)	35 (28.00%)	
Student	9 (6.98%)	6 (4.80%)	
Others	12 (9.30%)	16 (12.80%)	
Economic impression			0.572
High	20 (15.50%)	6 (4.80%)	
Medium	80 (62.02%)	72 (57.60%)	
Low	29 (22.48%)	47 (37.60%)	
Smoking habit			0.299
Non-smoker	120 (93.02%)	120 (96.00%)	
Smoker	9 (6.98%)	5 (4.00%)	
Residence area			0.847
Rural	83 (64.34%)	82 (65.60%)	
Urban	46 (35.66%)	43 (34.40%)	
Previous history of MDD			< 0.001
Yes	67 (51.94%)	0 (0.00%)	
No	62 (48.06%)	125 (100.00%)	
Family history of MDD			< 0.001
Yes	32 (24.81%)	0 (0.00%)	
No	97 (75.19%)	125 (100.00%)	

*Abbreviations*: BMI, body mass index; CED, chronic energy deficiency; MDD, major depressive disorder; SEM, standard error mean

### Clinical profiles and laboratory findings

**Fig. 1:**
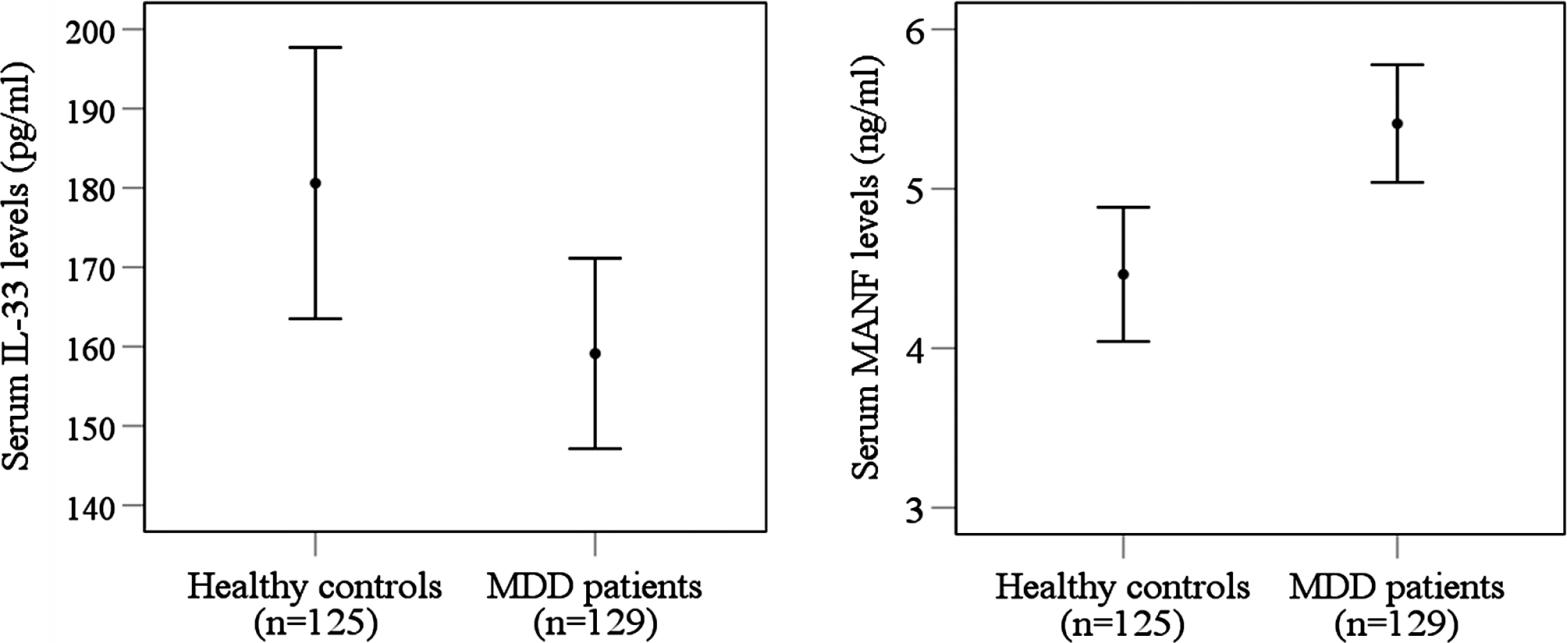
Comparison of serum interleukin-33 and mesencephalic astrocyte derived neurotrophic factor levels between MDD patients and healthy controls

**Fig. 2:**
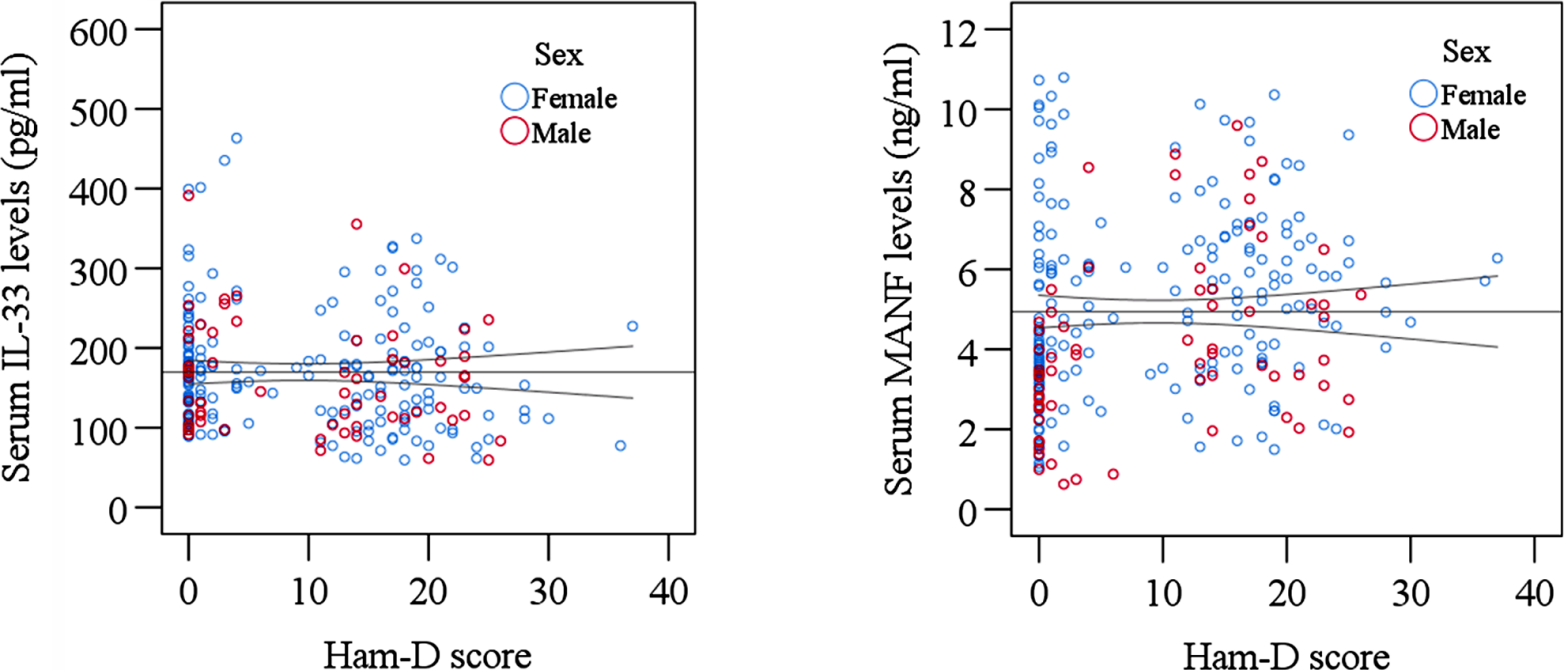
Sex-specific scatter plot graphs showing the association of IL-33 and MANF levels with Ham-D scores of study participants

The clinical outcome and laboratory findings of the study subjects are presented in Table 2. We observed lower serum IL-33 levels in MDD patients (159.12 ± 6.07 pg/ml) compared to HCs (180.60 ± 8.64 pg/ml) (p = 0.042). Compared to male MDD patients, female MDD patients had more significant changes in serum IL-33 levels. We also found higher serum levels of MANF in MDD patients (5.40 ± 0.19 ng/ml) than in HCs (4.46 ± 0.21 ng/ml) (p < 0.001). We also observed significantly higher mean serum MANF concentrations in male (4.99 ± 0.37 ng/ml) MDD patients compared to healthy male controls (3.13 ± 0.30 ng/ml). The alterations in serum IL-33 and MANF levels have been displayed graphically in Fig. 1. We observed the alteration of serum IL-33 and MANF with the severity scores in MDD patients from the sex-specific scatter plot graphs (Fig. 2). The best fit lines in Figure indicate the relationships among different data points. Still, there was no significant association between the severity of MDD and this alteration. We observed that depression was more severe in female MDD patients than in male MDD patients. Moreover, we didn’t observe any significant association between altered *serum IL-33 and MANF levels among the patients.*

**Table 2:** Clinical profile and laboratory findings of the study population

Parameters	MDD patients (n = 129) Mean ± SEM	Healthy controls (n = 125) Mean ± SEM	*p-value*
Age (years)	30.67 ± 0.86	30.18 ± 0.82	0.675
Male (P/C:34/33)	29.97 ± 1.80	30.88 ± 1.85	0.725
Female (P/C:95/92)	30.93 ± 0.98	29.92 ± 0.89	0.451
BMI (kg/m^2^)	23.44 ± 0.44	24.38 ± 0.37	0.105
Male (P/C:34/33)	23.78 ± 0.88	25.87 ± 0.68	0.066
Female (P/C:95/92)	23.32 ± 0.51	23.85 ± 0.43	0.430
Ham-D score	17.95 ± 0.44	1.00 ± 0.14	< 0.001
Male (P/C:34/33)	17.71 ± 0.77	1.00 ± 0.27	< 0.001
Female (P/C:95/92)	18.04 ± 0.53	1.00 ± 0.17	< 0.001
Serum IL-33 (pg/ml)	159.12 ± 6.07	180.60 ± 8.64	0.042
Male (P/C:34/33)	149.87 ± 11.30	169.94 ± 11.88	0.225
Female (P/C:95/92)	162.43 ± 7.18	184.42 ± 10.95	0.093
Serum MANF (ng/ml)	5.40 ± 0.19	4.46 ± 0.21	0.001
Male (P/C:34/33)	4.99 ± 0.37	3.13 ± 0.30	< 0.001
Female (P/C:95/92)	5.56 ± 0.21	4.94 ± 0.25	0.065

*Abbreviations*: BMI, body mass index; Ham-D, 17-item Hamilton depression rating scale; IL-33, interleukin-33; MANF, mesencephalic astrocyte derived neurotrophic factor; MDD, major depressive disorder; P/C, patients/control; SEM, standard error mean

## Discussion

In this investigation, we measured the serum IL-33 levels in MDD subjects and HCs. According to our analysis, IL-33 levels in MDD patients were significantly lower than in HCs. Few previous studies have tried to conclude the involvement of IL-33 in depression. Though it was observed that the serum levels of IL-33 were elevated not only in depressive patients but also in patients with other psychiatric disorders [31–33], some studies found an attenuated anti-inflammatory action of IL-33, which is aligned with our study finding of the lower level of IL-33 in patients [39, 40]. The role of inflammation in MDD has been observed, and inflammation has been revealed to be a critical factor in the development of depression [39, 41]. The inflammatory responses to depression are mediated by a wide variety of peripheral biomarkers. Oxidative stress markers, altered neurotrophic levels, a change in pro-inflammatory cytokine levels, neurotoxic metabolites of tryptophan degradation, etc., are some of them [42]. Cytokine-mediated immune activation is connected to the pathophysiology of depression by several mechanisms, including kynurenine pathway activation [41, 43], hypothalamic-pituitary-adrenal axis activation [42, 44], reuptake and release of neurotransmitters [45], hyperexpression of serotonin transporters [46], decreased neuronal growth factors [47], and neurodegeneration [48]. These studies might prove the involvement of IL-33 in depression, though the exact association could not be concluded.

On the other hand, this study also measured serum MANF levels. We found that serum MANF levels were significantly higher in MDD patients than in HCs. The differences in serum MANF levels between male subjects were also found to be statistically significant, while in the case of female subjects, such differences were not significant. These findings suggest that the role of MANF in causing depression is more prominent in men than women. Until now, the function of MANF in causing mental illness has not been thoroughly investigated in many studies. A study revealed that patients with Parkinson’s disease showed significantly higher levels of MANF than HCs [49]. Another study has demonstrated that MANF acts as a regulator of lipid metabolism [37]. A few studies in recent years have revealed that lipid abnormalities may be crucial to the pathogenesis of MDD [50]. So, MANF might have a role in connecting hypolipidemia with MDD [37]. A study concluded that low-density lipoprotein cholesterol (LDL-C) was found to be higher in MDD patients than HCs [51], and another study found higher serum MANF levels in patients with hyperlipidemia along with high levels of LDL-C [52]. So, there might be an association between high serum MANF level and the progression of depression. There hasn’t been enough investigation into how MANF affects depression, but its association with depression could be predicted from these study findings. Here, our investigation tried to correlate the serum concentration of MANF with MDD. According to our knowledge, this is the first study investigating serum IL-33 and MANF levels among Bangladeshi MDD patients and we observed altered serum levels of these biological markers were found to be associated with MDD.

Multiple previous studies and our own have demonstrated an association between MDD, the influence of pro-inflammatory cytokines, and elevated LDL-C levels [53–55]. Increasing the anti-inflammatory function of cytokines and decreasing LDL-C levels can, therefore, aid in treating MDD. When combined with other treatments for MDD, exercise can be highly effective. Physical activity can increase the anti-inflammatory activity of cytokines [56, 57] and decrease cholesterol levels, decreasing MANF levels. Consequently, physical activity may aid in achieving an optimal serum level of MANF and IL-33 and their desired action [40, 58–60], which will aid in treating and preventing MDD.

Studies investigating IL-33 and MANF in the serum of MDD patients are limited, and most of them have produced contradictory results. The serum IL-33 and MANF levels were analyzed in MDD patients and HCs under the same environmental conditions. Therefore, we anticipate the current investigation’s results will contribute to assess the risk of developing MDD. This study’s outcomes will help us comprehend the pathophysiology of MDD more appropriately. Besides, it will also assist in assessing the risk of depression. These findings may help psychiatrists to understand the pathophysiology of depression. Consequently, these altered markers will be helpful for clinicians as early risk assessment tools to evaluate depression risks.

## Limitations

The current study has a few limitations that should be considered. Firstly, the entire neuroinflammatory process of MDD cannot be accurately represented by measuring only IL-33 and MANF levels. Secondly, we did not consider the effect of lifestyle, dietary supplementation, or treatment on the analyzed parameters in the present investigation. In the same population, it would be appropriate to measure other parameters. Through this study, in individuals with MDD, we could not observe treatment responses or changes in IL-33 and MANF levels over time. To obtain better findings, we recommend additional research using more samples.

## Conclusion

Based on the results of the current investigation, associations between altered serum IL-33 and MANF levels and the pathophysiology of MDD have been noticed. MDD patients have reduced serum concentrations of IL-33 and elevated serum concentrations of MANF compared to HCs, according to this study. Hence, the altered serum IL-33 and MANF levels may indicate the development of MDD. However, based on the results of this study, we recommend further investigation to determine the impact of the aforementioned markers on depression using larger and more homogeneous samples.

## Data Availability

Data supporting our findings are available from the corresponding author on reasonable request.

## Abbreviations

BMI: Body mass index
BSMMU: Bangabandhu Sheikh Mujib Medical University
DSM-5: Diagnostic and statistical manual of mental disorders, 5th edition
ELISA: Enzyme-linked immunosorbent assay
Ham-D: Hamilton depression rating scale
HCs: Healthy controls
HPA: Hypothalamic-pituitary-adrenal
IL-33: Interleukin-33
MANF: mesencephalic astrocyte derived neurotrophic factor
MDD: Major depressive disorder
SPSS: Statistical package for the social sciences
